# A Comparison of Fresh and Frozen Lamb Meat—Differences in Technological Meat Quality and Sensory Attributes

**DOI:** 10.3390/ani12202830

**Published:** 2022-10-18

**Authors:** Elin Stenberg, Katarina Arvidsson-Segerkvist, Anders H. Karlsson, Aðalheiður Ólafsdóttir, Óli Þór Hilmarsson, María Gudjónsdóttir, Guðjón Thorkelsson

**Affiliations:** 1 Department of Animal Environment and Health, Swedish University of Agricultural Sciences, P.O. Box 234, 53223 Skara, Sweden; 2 Matís Ohf, Vínlandsleið 12, 113 Reykjavík, Iceland; 3 Faculty of Food Science and Nutrition, University of Iceland, Aragata 14, 101 Reykjavík, Iceland

**Keywords:** meat colour, cooking loss, Warner-Bratzler shear force, tenderness, odour, texture, flavour, LF-NMR, water and lipid distribution

## Abstract

**Simple Summary:**

Freezing is used to extend the storage time of meat and is common practice in lamb meat production, since it maintains a steady supply of seasonal meat throughout the year and allows shipping over long distances. Fresh meat may also be purchased and frozen at home, to enable longer storage of the product before consumption. Freezing is the best preservation method, apart from chilling of fresh meat. However, differences in quality parameters between fresh and frozen meat may influence consumer choice and preferences. It is thus important to evaluate these differences, and how they are affected by conditions and animal handling during primary production, slaughter method and storage conditions before retail sale. This study examined the effect of freezing on technological meat quality and sensory attributes in lamb meat samples collected at two different slaughterhouses using different slaughter methods. Several differences between fresh and frozen-thawed meat were detected in terms of technological meat quality and sensory attributes, including colour, Warner-Bratzler shear force, cooking loss, flavour attributes and juicy texture.

**Abstract:**

Technological meat quality and sensory attributes of fresh and frozen lamb meat were compared. Samples were collected from two abattoirs (one small-scale, one large-scale) that use different slaughter methods in terms of chilling regime and electrical stimulation. The fresh and frozen meat samples included products from both slaughter systems. Ten twin pairs of ram lambs were used in the study, with one of each twin slaughtered at each abattoir. Fresh meat was analysed after chilling and frozen meat was stored frozen for three months and analysed after thawing. The *Musculus longissimus thoracis et lumborum* was analysed for colour, cooking loss, sensory attributes, Warner-Bratzler shear force (WBSF) and distribution of water and lipid within each meat sample. Meat samples analysed after frozen storage were darker, less red and more yellow than the fresh meat. Freezing and frozen storage increased fluid loss and WBSF compared with the fresh meat, due to protein denaturation. Frozen storage affected sensory attributes by increasing fatty odour, frying flavour, sour flavour, fatty flavour and liver flavour, and by reducing juicy texture and mushy texture.

## 1. Introduction

Lamb meat is traditionally regarded as a seasonal meat product in the Northern Hemisphere, due to the seasonal availability of the meat. The traditional lambing season is in the spring, due to the usual reproduction cycle of the ewe in temperate regions [1]. Hence, most of the lamb meat in northern hemisphere regions becomes available in autumn, and any surplus meat not sold fresh can be stored as frozen until the market for fresh meat has declined. Freezing of meat has given markets an opportunity to offer sales of lamb all year round, which can result in a better market price for lamb meat. Furthermore, the surplus product does not have to be sold off at low prices when supply exceeds demand for fresh meat in the intensive slaughter season [2]. Therefore, freezing of meat can be used to stabilise the market and increase product flexibility [3,4], allowing producers to sell the meat at a better price and making lamb meat available to consumers all year around [5]. Meat is stored frozen to preserve the product and to keep the meat quality as high as possible [6]. The impact of freezing on meat quality has been reviewed by Leygonie et al. [7], who concluded that freezing has well-documented effects on moisture loss in meat, but that the literature is inconsistent about the combined effects of freezing and thawing on other parameters, such as colour and tenderness. Studies have also reported increased moisture losses with increasing frozen storage time in both lamb [8] and beef [9]. However, the effect of frozen storage on the sensory properties of lamb meat has been shown to be small when assessed by a trained panel, and should not affect consumer acceptance for the product [10]. Differences between fresh and frozen meat have been reported when untrained consumer panels assessed lamb meat for tenderness, flavour and overall acceptability [10,11]. When studying differences between fresh and frozen meat, it is important to consider how the freezing process affects meat quality attributes. For example, Luyet [12] showed that ice crystals are formed differently depending on whether the freezing is fast or slow. When the rate of freezing is slow, ice crystals form in large bundles between muscle fibres, whereas during rapid freezing ice crystals form both within and between cells [12]. Petrović et al. [13] observed a clear increase in cooking loss for both fast and slow freezing rates compared with unfrozen meat. In that study, rapid freezing procedures resulted in more intracellular ice crystals that were smaller in size, which thereby reduced the weight losses (thawing and cooking) of the meat compared with slower freezing procedures [13]. Differences in crystal formation contribute to the fluid loss after thawing and cooking, due to more or less disruption to the fibre structure, which enables water to leave the muscle cells [12]. This water cannot be rebound to proteins during the thawing process, and is hence lost as thawing loss [13]. Freezing has been shown to improve shear force values [14] or to cause no detectable differences in lamb meat [11]. However, the combined effect of chilling and frozen storage has not been fully evaluated according to a review by Coombs et al. [15], with few studies in particular analysing the effects of longer chilling storage combined with frozen storage exceeding 3–4 months. The aim of the present study was therefore to evaluate the combined effect of chilling and freezing storage, and whether frozen lamb meat displays differences in meat quality attributes compared with fresh meat. The hypothesis was that regardless of chilling regime, freezing would lead to a higher fluid loss which influences meat quality.

## 2. Materials and Methods

A detailed description of the experimental design, including animals, slaughter systems, sample treatments and experimental analyses, is provided in a companion paper by Stenberg et al. [16]. In brief, 10 pairs of intact ram lamb twins originating from the same farm and of the Icelandic sheep breed were used in this study. Ten lambs (one from each pair) were slaughtered at a large-scale abattoir and the remaining 10 were slaughtered at a small-scale abattoir. Both abattoirs kept the lambs in lairage for 10–12 h overnight before slaughter. During lairage, all animals had access to water, but no feed. All lambs were on average 160 days old at slaughter and had an average hot carcass weight of 19.8 ± 1.4 kg (mean ± st. dev.). All animals were hung by the Achilles tendon after dressing. Under Icelandic legislation (Slaughter Regulation Act 461/2003, Animal Welfare Act 55/2013, Quality Controlled Sheep Farming Act. 1160/2013), no ethical approval was needed before execution of the study.

### 2.1. Slaughter Facilities

Ten lambs were slaughtered at a small-scale abattoir slaughtering about 75 lambs/day, and the other 10 were slaughtered at a large-scale abattoir slaughtering 2500 lambs/day. The small-scale abattoir used captive bolt stunning and kept the carcasses at 10–15 °C during the first six hours after slaughter, followed by chilling at 3–4 °C for 30 h, at which point samples of *M. longissimus thoracis et lumborum* (LTL) were removed. The large-scale abattoir used electrical stunning and applied electrical stimulation to all carcasses prior to entering the chiller, and the carcasses were hung for 30 h at 2–4 °C before sampling of LTL.

### 2.2. Sampling, Packaging and Handling of LTL Muscle

Both LTL muscles, including subcutaneous fat, were removed from all carcasses at the location between the last lumbar vertebra and the seventh thoracic vertebra. The LTL muscle from the left side was labelled as fresh and the muscle from the right side was labelled as frozen. All muscle samples were vacuum-packed in 25 cm × 35 cm bags. Two samples from the large-scale abattoir were unfortunately frozen by mistake and could not be analysed as fresh meat. Each LTL muscle, after removal of subcutaneous fat, was divided into parts to provide samples for the different analyses. Colour and NMR analyses were done on uncooked meat, and sensory analysis and WBSF were done on cooked meat. The cooking procedure was standardised as sous vide cooking for one hour (Anova Precision Cooker, Anova Culinary Inc., San Francisco, CA, USA) at 68 °C, and then flash-frying at a high temperature on each side for 30 s. Slices of 1.0 cm of the most posterior part of uncooked *M. longissimus lumborum* (LL) were used for NMR samples. Slices of 3.0 cm of cooked anterior par of *M. longissimus thoracis (LT)* were used for WBSF samples and the remaining part of LL was used for sensory analysis.

### 2.3. Fresh Samples

The fresh samples were stored at 2–4 °C and aged for 6–7 days before analysis.

### 2.4. Frozen Samples

The frozen samples were subjected to two different procedures after slaughter, based on standard operations at the two different abattoirs. The samples from the small-scale abattoir were aged for four days at 2 °C before samples were frozen at −24 °C and stored for three months. The samples from the large-scale abattoir were frozen on the day after slaughter, and then kept at −24 °C for three months. All frozen samples were thawed at 4 °C overnight before analysis.

### 2.5. Colour

Colour measurements were carried out on the fresh meat before vacuum packaging and cooking, and on the frozen meat after vacuum packaging and thawing. Each sample was allowed to bloom for one hour at 20 °C before the colour was measured in triplicate for each muscle sample. All samples were analysed with a Minolta CR-300 colorimeter (Konika Minolta, Tokyo, Japan) with a D65 light source. The colour measurements provided information about the lightness (L* value), redness (a* value) and yellowness (b*) of the muscle samples.

### 2.6. Thawing and Cooking Loss

All fresh samples were weighed before and after the cooking process, to calculate the loss after cooking (%). The frozen samples were weighed as frozen and after cooking, to calculate the combined loss of fluid from both thawing and cooking.

### 2.7. Warner-Bratzler Shear Force (WBSF)

Muscle tenderness was measured instrumentally on cooked samples of LTL by a Warner-Bratzler knife (TA-7) comprising a guillotine block coupled to a texture analyser (TA.HD Plus Connect, Godalming, Surrey, UK), which moved at a speed of 2.5 mm/second. The sample size for analysis was 1.0 cm × 1.0 cm × 3.0 cm (width × height × length). Samples were cut orthogonally to the muscle fibre direction, and each WBSF value was derived from quadruple measurements for each muscle sample.

### 2.8. Sensory Evaluation

A trained panel (generic descriptive analysis) [17] consisting of 6–10 panellists participated in each of a total of five testing occasions, depending on the availability of individuals. Each sensory session evaluated four different samples. To check panel performance and the performance of the individual assessors, PanelCheck V1.4.0 (Nofima, Tromsö, Norway) software was used. In each session, each panellist was given one 2 cm thick sample from the same location within each LTL muscle, which means that each LTL was divided into 10 different test samples. Samples were still warm when presented to the panellists in individual aluminium containers for each sample. The test samples were numerically coded with randomised numbers and were presented randomly to the panellists. Each panellist carried out their testing in a separate cubicle with standardised light, where they were given crackers and water to be consumed between samples to avoid residual flavour contamination. The evaluation was carried out for the following sensory traits: odour (frying, sour, fatty and liver), flavour (frying, sour, fatty, sweet and liver) and texture (softness, tenderness, juiciness and mushiness), which were rated on a linear scale from 1 to 100 for each trait. Before the actual test the panellists were calibrated at two panel training sessions to select and define the same specific characteristics and intensity of each individual sensory attribute. The defined sensory attributes to describe odour, appearance, flavour and texture were then used in the study to evaluate the meat. A more detailed description of the attributes can be found in Table S1 in the Supplementary Materials.

### 2.9. Low Field Nuclear Magnetic Resonance (LF-NMR)

Samples of the fresh meat and frozen-thawed uncooked meat were analysed by low field nuclear magnetic resonance (LF-NMR) at ambient temperature (20 ± 1 °C). A Minispec mq 20 benchtop NMR analyser (Bruker Optics, Rheinstetted, Germany) was used to analyse the water and lipid distribution in the meat muscle through transversal relaxation time analysis. Three samples (approximately 1 g) were cut from each muscle, transferred individually to 10 mm NMR sampling tubes and placed inside the magnet. The Carr-Purcell-Meibook-Gill (CPMG) pulse sequence [18,19] was applied, with an interpulse spacing of 100 µs, 8000 echoes, a recycle delay of 10 s and 16 repetitive scans. NMR relaxation time data were collected using Bruker Minispec software (Bruker Optics, Rheinstetten, Germany) and then further analysed in Matlab (Mathworks Inc., Natric, MA, USA).

### 2.10. Statistical Analyses

Technological meat quality and sensory data (Tables S2 and S3) was analysed by Proc Mixed in the Statistical Analysis Software (SAS) [20], with twin pair of lambs as random effect, in the model:

Model: Y_ijk_ = µ + S_i_ + F_j_ + SF_ij_ + P_k_ + e_ijkl_

where Y_ijk_ is the dependent variable, µ is the grand mean, S_i_ is the fixed effect of slaughter system, F_i_ is the fixed effect of fresh or frozen (sample treatment), SF_ij_ is the interaction between slaughter system and sample treatment, P_k_ is the random effect of twin pair, and e_ijkl_ is the residual error.

A general Satterwaite approximation for the denominator degrees of freedom was performed, using the SATTERTH option in SAS. Differences were considered significant at *p* ≤ 0.05, and marginal significance was assumed at 0.05 < *p* ≤ 0.10.

The relaxation data (Tables S4 and S5) were maximum-normalised prior to further analysis. The normalised data were analysed by principal component analysis (PCA) in Unscrambler X^®^ (Camo AS., Oslo, Norway), and then fitted to a multi-exponential curve using the Low-field NMR toolbox for Matlab, as described by Pedersen et al. [21]. PCA was performed in Unscrambler X (Camo AS, Oslo, Norway) on all data to find similarities and differences between samples from the two treatments. The data were centred and weighted with the inverse of the standard deviation of each variable, to correct for the use of different units.

Results presented directly in the text below are least squares means ± standard deviation.

## 3. Results

### 3.1. Technological Meat Quality Attributes

An effect of freezing compared with fresh meat was found in colour measurements, cooking loss and WBSF (Table 1). There was a tendency (*p* = 0.0654) for a significant difference in lightness (L*) between fresh and frozen meat, where the fresh meat was lighter, i.e., had higher L* value, than the frozen meat (Table 1). A difference (*p* = 0.0080) was also found in redness (a*), where the fresh meat was redder, i.e., had a higher value than the frozen meat (Table 1). The parameter cooking loss also showed a significant difference (*p* = <0.0001), with the frozen meat displaying a greater loss than the fresh meat (Table 1). There was a tendency for significance in WBSF, where the frozen meat had a higher shear force value than the fresh meat (Table 1).

**Figure 1:**
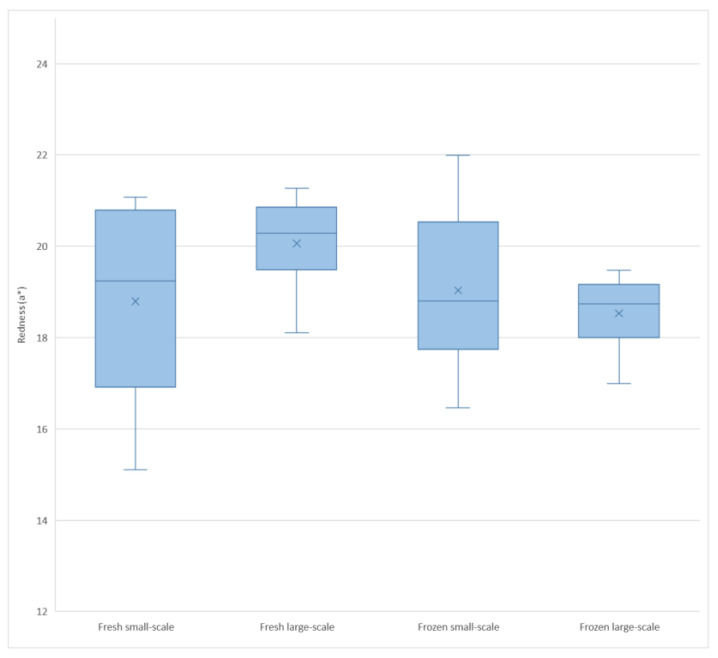
Boxplot showing redness (a*) for fresh and frozen meat samples from the small-scale and large-scale abattoirs.

**Figure 2:**
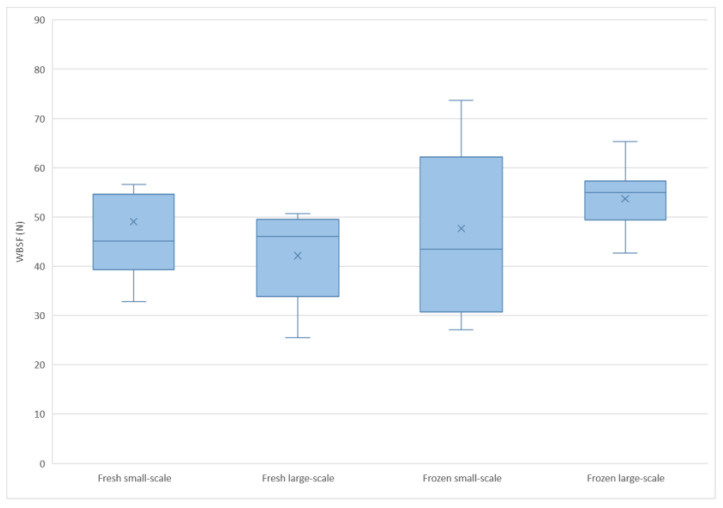
Boxplot showing Warner-Bratzler shear force (WBSF) in Newtons (N) for fresh and frozen meat samples from the small-scale and large-scale abattoirs.

Interactive effects were found for redness (a*) and WBSF. There were two interactions between slaughter system and meat treatment (fresh or frozen) on redness (a*) (*p* = 0.0011) and WBSF (*p* = 0.0269). The interactive effect on redness comprised an increase in redness in frozen (a* = 19.0) compared with fresh (a* = 18.8) meat for the small-scale abattoir and a decrease in redness in the frozen (a* = 18.5) samples compared with fresh meat (a* = 20.2) for the large-scale abattoir (Figure 1). The WBSF value from the small-scale abattoir decreased in frozen (47.6 N) compared to fresh (49.0 N) meat. However, an increase in WBSF was found in frozen (53.7 N) compared to fresh (43.4 N) meat from the large-scale abattoir (Figure 2).

### 3.2. Sensory Attributes

For some of the specific odour, flavour and texture attributes tested, an effect of freezing was observed (Table 2). A significant difference (*p* = 0.0229) was observed for the attribute fatty odour, where frozen meat had a higher score than fresh meat (Table 2). The attribute frying flavour was significantly (*p* = 0.0340) higher in the frozen than the fresh meat (Table 2). Similar differences were seen in the attributes sour flavour (*p* = 0.0204), fatty flavour (*p* = 0.0003) and liver flavour (*p* = 0.0222), which all showed higher scores for the frozen meat than the fresh (Table 2). There was also a difference in terms of juicy texture (*p* = 0.0001) and a tendency for a significant difference in mushy texture (*p* = 0.0714), with both attributes showing higher scores for fresh meat compared with frozen meat (Table 2).

### 3.3. LF-NMR Analysis

**Figure 3:**
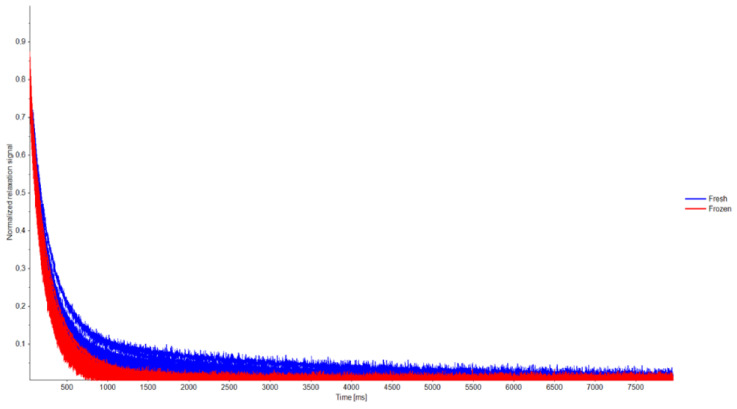

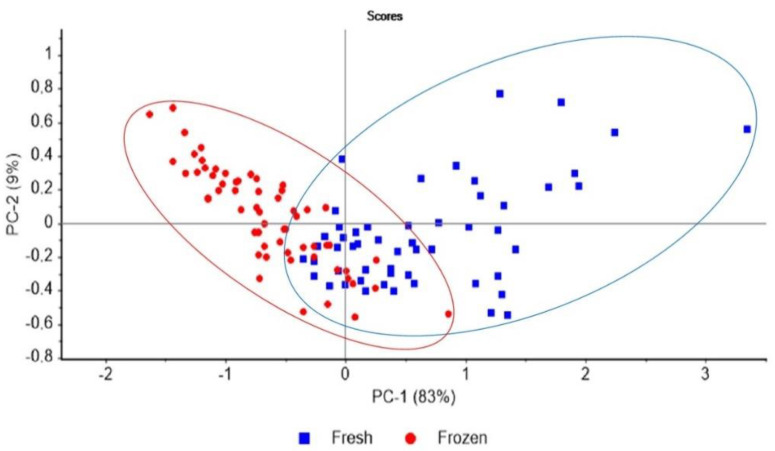
(**upper panel**) Normalised transversal relaxation curves obtained with LF-NMR for fresh and frozen lamb meat samples, and (**lower plot**) principal component analysis (PCA) plot of these relaxation curves. The blue ellipse indicates the grouping of fresh meat samples, and the red ellipse indicates frozen meat samples.

The transverse relaxation time curves were collated and analysed by PCA, to identify sample similarities and variations in water and lipid characteristics and their distribution throughout the muscle samples (Figure 3). The first two principal components in the PCA plot explained 92% of the variation between samples. Sample groupings showed a clear distinction between fresh and frozen lamb meat samples (Figure 3). A more confined cluster of the frozen samples compared with the fresh samples indicated that the between-sample variation in water and lipid distribution and characteristics was smaller after freezing than in the fresh meat samples.

To explain the effects of freezing and to identify potential differences in water distribution in the meat samples, the relaxation time data were fitted to a multi-exponential curve. Proton relaxation analysis identified three proton populations in the meat samples (Table 3). In agreement with earlier LF-NMR studies on muscle-based foods [22,23,24], the origin and allocations of these proton populations were interpreted as follows: the proton distribution indicated the presence of a small water population A_2b_ (approximately 10% of all water) with translational relaxation parameter T_2b_ of approximately 10–16 ms in all meat samples (Table 3). This population is believed to relate to water molecules in close association with macromolecules in the meat. A dominant population A_21_, corresponding to approximately 80% of the identified protons, with an approximate relaxation time of 40–50 ms was observed in all meat samples. This population mainly correlates to restricted water in myofibrillar cells and lipids within the muscle. Finally, a third population T_22_ was observed, contributing to 7–14% of the protons, with relaxation times in a wider range. This population is believed to correspond to less restricted water within the muscle, or extra-myofibrillar water [22,23,24].

Correlation analysis revealed medium negative correlations between the relaxation times and fluid loss (*r = −0.532* and *r = −0.598* to T_21_ and T_22_, respectively) and medium positive correlations between the extra-myofibrillar water proportion A_22_ and fluid loss (*r = 0.466*). Correlation analysis of the LF-NMR and textural and sensory data revealed medium negative correlations between T_2b_ and soft (*r = −0.539*), and tender texture (*r = −0.496*).

## 4. Discussion

This study evaluated differences between fresh and frozen lamb meat in terms of technological meat quality and sensory attributes. The decision to include meat from large-scale and small-scale slaughter treatments in the fresh and frozen groups was based on common practice in current commercial production. This is therefore the most accurate way to assess how meat available on the market could be affected by freezing and frozen storage. The post mortem handling of samples before freezing differed between the two slaughter systems covered by the study. These differences in post-slaughter treatment could have had an effect on quality characteristics such as colour stability and water loss, based on findings by Choe et al. [25] in a study investigating the effect of ageing meat at different temperatures and for different periods before freezing. However, no such differences were detected in this study.

### 4.1. Fluid Losses

Fluid losses occurred, as combined thawing and cooking losses, and affected several sensory and physicochemical attributes, such as juicy texture. Fluid losses and juicy texture were both affected by freezing, with the frozen samples showing greater losses after thawing and cooking and perceived to be less juicy by the sensory evaluation panel. The sensory attribute juicy texture is discussed below together with the other sensory attributes. The literature describes different types of fluid losses, such as drip loss, thawing loss, cooking loss, fluid loss, water loss or changes in water-holding capacity (not defined further) or combinations of two or several of these parameters [8,9,26,27,28,29]. In the present study, losses of fluids were measured as combined thawing and cooking losses, although the individual effect of these parameters could be examined in future studies in order to distinguish between the fractions. The results in the present study are compared with those of previous studies reporting any type of loss of fluids, or not, in relation to freezing compared with unfrozen meat. Previous studies have shown that freezing can have an increasing effect on fluid losses from lamb meat [8,29], which is supported by the results in the present study. Similar findings have been reported for beef meat, by Vieira et al. [9] observing increased fluid losses as a results of freezing. Muela et al. [29] did not find differences in cooking loss between fresh and frozen meat, with the explanation that differences may have occurred in thawing losses instead of cooking losses. The thawing losses were greater for the frozen compared with the unfrozen meat samples in that study, and hence the total fluid losses were greater for the frozen samples [29]. Bhattacharya et al. [27] ascribed drip loss to protein denaturation by the high ionic strength of extracellular fluid as an effect of freezing, resulting in the protein losing its water-holding capacity [27,29]. Since protein denaturation affects water-holding capacity negatively, this may be an explanation for the increased fluid loss observed after freezing in the present study. Another explanation could be that ice crystallisation, which occurs when meat is frozen, can damage cell structures and lead to increased fluid losses when the meat is thawed [12,13].

### 4.2. Colour

When consumers are choosing meat in the supermarket, meat colour is one of the most important quality measures they consider. In particular, the redness (a* value) is often identified as the most important measure, since consumers often associate a certain degree of redness with meat that is safe to eat [30]. However, whether red colour can be used as the single most valid method for evaluating safe meat has not been established within the scientific community. The results in this study revealed a change in colour as an effect of freezing, as well as an interactive effect between slaughter systems and sample treatment (fresh/frozen) on redness. Comparing meat from the fresh and frozen groups, the frozen and thawed samples tended to have darker, less red and more yellow colouring than the fresh meat. An effect of freezing on meat colour has been described in previous research [10,25,29,31]. The decrease in lightness (L*) in the thawed meat compared with the fresh in our study supports previous findings [29,31,32]. Farouk et al. [28] attributed this reduction in L* to higher thaw drip in slowly frozen beef samples, leading to greater light reflection and thereby a lighter colour compared with fast-frozen beef samples. Since the frozen meat in the present study had greater fluid losses after cooking, the explanation suggested by Farouk et al. [28] may be valid also for our results. The observed reduction in redness (a*) might be explained by reduced activity of metmyoglobin-reducing enzymes, leading to accumulation of metmyoglobin that was visually apparent as reduced redness in the frozen samples compared with the fresh [32]. The decrease in redness (a*) might therefore be due indirectly to the loss of fluids in the frozen samples after thawing and cooking. However, differences in redness (a*) are not always found between fresh and frozen meat, e.g., Muela et al. [29] did not find differences in redness (a*) between fresh and frozen meat when looking at different freezing methods and durations of frozen storage. The results from that study suggest that redness (a*) is not exclusively affected by freezing, a suggestion supported by Fernández et al. [33] who claim that freezing protects meat from decolouration, resulting in recovery of meat colour after thawing and thereby no visible differences in meat colour. When meat is less light (L*) and less red (a*), the yellow colour (b*) may be more prominent, and the increase in b* in our frozen samples could therefore be an indirect effect of the decrease in redness (a*). Another theory is that increased yellowness (b*) is due to oxidation and yellowing of fat, as suggested by Moore and Young [30] on examining the effect of storing meat under display film in terms of e.g., discolouration. The intramuscular fat content in the lamb meat analysed in this study was not measured, so we offer this as a speculative theory, rather than an explanation. Variation in yellowness (b*) in pork muscle has previously been described as an effect of variation in fractions of deoxymyoglobin and oxymyoglobin, as well as an effect of internal reflectance [34]. Since there was a change in both lightness (associated with internal reflectance) and redness (associated with the different myoglobin forms) between fresh and thawed samples in this study, this could explain the difference in yellowness. However, the numerical differences in L*, a* and b* between the fresh and frozen sample groups were quite small, and should have only a low impact when consumers are making an assessment with the human eye before prospective purchases. The differences in meat colour may affect consumer acceptance of lightness and redness, according to previous research [34]. Khliji et al. [35] found that when redness (a*) is ≥9.5 and lightness (L*) is ≥34, consumers would regard meat colour as acceptable based on colour parameters. The significant differences seen in the colour parameters examined in the present study lead us to conclude that there were differences between the fresh and frozen meat samples, but the small numerical differences indicate that these differences were not undesirable in either treatment from a consumer perspective. The same conclusions can be drawn on examining the effect of the interactive difference in redness. The differences in ageing between the two slaughter systems tested could have affected colour stability, as seen in previous work [30]. However, the numerical differences in redness between slaughter systems and sample treatments were small, and should not pose a risk of consumers reacting negatively when visually assessing the meat in a purchase situation.

### 4.3. WBSF

There was a tendency for a difference in WBSF between the fresh and frozen meat, with the frozen meat having a higher value. Previous research has found contrasting effects on WBSF on comparing frozen meat with fresh. For example, Duckett et al. [36] did not detect any difference in shear force (SF) between fresh and frozen meat from normal lambs, but observed a decrease in SF following freezing for meat from lambs with the callipyge gene. In contrast, Smith et al. [6] found an increase in SF in frozen meat compared with fresh and Muela et al. [31] observed an increase in WBSF in meat after frozen storage for 15 or 21 months compared with fresh meat. However Muela et al. [31] observed no difference in WBSF in meat stored frozen for one or nine months compared with fresh meat [31]. Another study reported a decrease in WBSF values as an effect of increased frozen storage [9]. Hence, it may not be correct to state unequivocally that freezing and frozen storage increases or decreases WBSF, since many factors influence the outcome. Such factors can include frozen storage duration [31], freezing rate [3], and a combination of ageing before frozen storage and storage time [9]. With this in mind, it may not have been the freezing and thawing itself that affected the WBSF in frozen meat compared with fresh in this study. The effect seen in the present study was a tendency to significance for WBSF, which did not correspond to the results of the sensory testing where the panellists did not detect a difference between fresh and frozen meat in the attribute tender texture. It can therefore be concluded that the tendency to significance for WBSF may not have been detectable by consumers, which could be seen as a positive result when evaluating the freezing of meat.

Since WBSF is a method for evaluating tenderness, it is important to reflect on the numerical values obtained when discussing the results. As shown in Figure 2, all mean values of WBSF were higher than 40 N and some even over 50 N. Previously published work has suggested a threshold of ≤5 kg (~49 N) for meat to be considered acceptably tender [37]. With this information in mind, it is reasonable to assume that all meat samples from the present study except the frozen samples from the large-scale abattoir would be rated acceptably tender from a WBSF perspective. It could also be stated that the WBSF results indicate that all meat samples within the present study are in the upper range, or above the threshold for acceptably tender meat. The differences detected for WBSF were not reflected in the sensory attribute texture tenderness, for which the sensory panel did not find a difference between fresh and frozen meat samples. This could be interpreted as a broader tolerance than 49 N, since the mean WBSF value was 46 *±* 13.1 N for fresh and 51 *±* 13.0 N for frozen meat.

The interactive effect on WBSF, with an increase in WBSF after freezing in meat from the large-scale abattoir compared with fresh meat and a decrease in WBSF after freezing in meat from the small-scale abattoir compared with the fresh samples, may have been influenced by differences in the ageing regime before freezing the meat. Meat from the small-scale abattoir was aged for four days before frozen storage and for six days before the fresh meat was tested. In comparison, meat from the large-scale abattoir was aged for one day before frozen storage and six days before the fresh meat was tested. This difference in ageing may partly explain the differences in texture between the abattoirs. The difference in WBSF between frozen and fresh meat was greater for the large-scale abattoir, and might be explained by the shorter ageing time. The reason for using different ageing regimes in the different abattoirs was, as previously mentioned, to mimic current production and enable valid comparison. It is thus important to note that increased ageing time before freezing in the large-scale abattoir could improve meat quality in terms of WBSF values.

### 4.4. Sensory Attributes

Quite a few of the sensory parameters analysed were affected by freezing in the present study. The overall explanation for the increases in sensory properties, where flavour accounted for most attributes affected, could be loss of fluid after thawing and cooking of frozen meat. An explanation for the decreased juiciness scores proposed by Bueno et al. [38] is that freezing causes loss of water owing to disruption of cell structures, resulting in higher concentrations of flavour (in this study represented by frying, sour, fatty and liver) compounds in the samples. The decreased mushiness scores for the frozen samples may also have been an effect of the decreased juiciness due to loss of fluids. Another explanation relates to increased firmness and release of flavour compounds while chewing. According to Pouliot et al. [39], decreased firmness makes the chewing process shorter, which results in less release of flavour compounds. Since the WBSF values were quite high (51 *±* 13.0 N) for the frozen group and there was also a decrease in juiciness, it may be reasonable to assume that more chewing was required for these samples, increasing the release of flavour compounds, which may be the reason for elevated flavour scores of the thawed meat in this study. However, there were no differences in the sensory attributes of tenderness and softness between the samples, which could contradict the previous reasoning. Overall, the decreased juiciness may have caused the increase in flavour attributes in the present study, despite not influencing the scores for softness and tenderness in the frozen samples. Further, the sensory panellists could not detect any differences in the colour of the cooked meat, which could also be connected to the small numerical differences obtained for the colour measurements of lightness, redness and yellowness. The sensory attribute fatty odour increased in the frozen samples compared with the fresh, possibly due to lipid oxidation during frozen storage, supporting findings by Muela et al. [31] of an increase in lipid oxidation with increased storage time up to 15 months. Lipid oxidation was not measured in the present study, however, so the cause of the increased fatty odour for frozen samples cannot be established with certainty. It is possible that a form of lipid oxidation accrued during storage in the frozen samples and resulted in the increase in fatty odour when assessing the samples for sensory attributes. Other studies focusing on sensory properties of fresh and frozen lamb meat have found that most attributes are unaffected when the meat is assessed by a trained sensory panel [10,11]. The only difference found in previous work was for tenderness scores, which increased for nine months of storage compared with fresh meat and meat frozen for 1, 15 or 21 months [10]. The lack of effect on tenderness and juiciness in previous work was attributed to relatively fast freezing rates and also to the absence of temperature fluctuations during storage [11]. This theory is supported by findings on the effect of fluctuating temperatures during frozen storage, with negative effects in terms of rancidity if meat is stored frozen at higher temperatures (−5 or −10 °C) before the ultimate freezing temperature (−35 °C) is established [40]. Both Muela et al. [10] and Muela et al. [11] concluded that the lack of differences between fresh and frozen lamb meat in terms of sensory attributes means that consumers should not be concerned about buying frozen or thawed meat due to decreased sensory quality. Although freezing influenced several sensory attributes in the present study, this does not mean that the differences were negative (or positive) from a consumer point of view.

### 4.5. LF-NMR Analysis

Some minor shifting trends between the proton populations were observed in the fresh and frozen meat. However, the only statistically significant difference in A_2i_ parameters was an increase in A_22_ after freezing the samples, indicating that the proportion of water within the extra-myofibrillar space of the muscle increased during freezing. Further, the frozen storage increased restriction of water, as indicated mainly by the sharp decrease in the T_22_ relaxation time parameter, corresponding to extra-myofibrillar water. This indicates that freezing led to a proportional shift in water from the myofibrillar space to the extra-myofibrillar cells, but also that restriction of the extra-myofibrillar water increased during frozen storage.

An increase in the transverse relaxation rate (R = T_2_^−1^) has been shown to correlate well with heat-induced protein denaturation of whey proteins [41], and with protein aggregation and denaturation during cooking of shrimp [42]. The observed decrease in relaxation times (T_21_ and T_22_) in this study thus indicates that the lamb muscle was partially degraded or denatured during frozen storage, and that the T_22_ parameter was especially sensitive to frozen storage-induced changes in muscle structure. Pearson’s correlation analysis showed medium negative correlations between the relaxation times and cooking loss, and medium positive correlations between the extra-myofibrillar water proportion A_22_ and cooking loss, indicating that freezing had a reducing effect on the water-holding ability of the muscle. This agrees with findings by Straadt et al. [22] that cooking induces shrinkage of myofibrils and a concurrent increase in the extra-myofibrillar space due to heat-induced expulsion of water from the myofibrillar matrix. This could also be linked to the increase in fluid loss after frozen storage.

Correlation analysis of the LF-NMR and textural and sensory data showed medium negative correlations between T_2b_ and soft and tender texture as assessed during the sensory evaluation. However, no such correlations were seen between the NMR parameters and the instrumental texture analysis (WBSF), indicating that meat texture was not highly affected by the water distribution of the muscle, potentially due to the small variation in textural properties in the samples assessed in the study.

## 5. Conclusions

To freeze meat may cause a negative effect on several meat quality parameters. It would therefore be valid to recommend usage of fresh meat to avoid negative effects on meat quality caused by freezing. The practical usage of freezing to increase storage time of meat does however promote freezing as a method to use both today and in the future. It is therefore of utter importance to further study the effect of freezing of meat and how to optimize the procedures of frozen storage to promote a low negative impact on meat quality.

## Figures and Tables

**Table 1:** Colour parameter values, cooking loss and shear force values (WBSF) of lamb meat tested fresh and after frozen storage for three months (mean ± standard deviation).

Parameter	Fresh, n = 18	Frozen, n = 20	SEM ^1^	*p*-Value ^2^
Lightness (L*)	37.5 ± 1.7	36.7 ± 1.4	0.38	0.0654
Redness (a*)	19.5 ± 1.8	18.8 ± 1.4	0.37	0.0080
Yellowness (b*)	4.5 ± 1.2	6.0 ± 1.4	0.33	0.0006
Fluid loss (%)	15 ± 3.1	27 ± 3.3	0.70	<0.0001
WBSF (N) ^3^	46 ± 13.1	51 ± 13.0	3.03	0.0837

^1^ Standard error of the mean. ^2^ Differences considered significant at *p* < 0.05 and tending towards significance at 0.05 < *p* ≤ 0.10. ^3^ Warner-Bratzler shear force measured in Newtons.

**Table 2:** Results of sensory analyses comparing muscle samples from fresh meat and meat after frozen storage for three months.

Parameter		Fresh, n = 18	Frozen, n = 20	SEM ^1^	*p*-Value ^2^
Odour attributes ^3^	Frying	33 ± 8.7	36 ± 6.4	1.78	0.2465
	Sour	12 ± 3.8	14 ± 2.9	0.82	0.1043
	Fatty	27 ± 3.2	32 ± 7.0	1.38	0.0229
	Liver	30 ± 4.6	32 ± 4.4	1.05	0.1665
Appearance attribute ^3^	Colour	31 ± 4.7	30 ± 6.8	1.44	0.4780
Flavour attributes ^3^	Frying	24 ± 4.5	29 ± 6.8	1.33	0.0340
	Sour	25 ± 5.1	31 ± 8.0	1.55	0.0204
	Fatty	16 ± 3.5	22 ± 4.8	0.98	0.0003
	Sweet	9 ± 1.6	9 ± 1.8	0.40	0.6240
	Liver	41 ± 5.2	45 ± 5.3	1.38	0.0222
Texture attributes ^3^	Soft	50 ± 13.9	53 ± 13.8	3.22	0.5586
	Tender	48 ± 16.0	50 ± 14.8	3.71	0.6816
	Juicy	49 ± 9.6	35 ± 11.2	2.40	0.0001
	Mushy	16 ± 4.7	14 ± 3.7	1.00	0.0714

^1^ Standard error of the mean. ^2^ Differences considered significant at *p* ≤ 0.05. ^3^ Sensory attributes were scored on a scale from 0–100, with 100 being the highest score.

**Table 3:** Transverse relaxation times (T_2i_, i = [b, 1, 2]) and apparent proton populations (A_2i_,) obtained through multi-exponential fitting of the LF-NMR relaxation data for the fresh and frozen lamb meat samples (mean ± standard deviation; n = 18 for fresh, n = 20 for frozen).

Parameters	Fresh, n = 18	Frozen, n = 18	SEM ^1^	*p*-Value ^2^
*Translational relaxation parameters*				
T_2b_ (ms)	13.6 ± 6.9	12.1 ± 8.8	1.92	0.5972
T_21_ (ms)	48.7 ± 4.3	41.0 ± 4.3	1.05	<0.0001
T_22_ (ms)	224.1 ± 89.3	105.5 ± 20.4	15.8	<0.0001
*Water population within muscle sample*				
A_2b_ (%)	9.1 ± 7.5	9.0 ± 14.5	2.80	0.9657
A_21_ (%)	81.5 ± 7.2	78.0 ± 13.2	2.59	0.3549
A_22_ (%)	9.4 ± 2.1	13.0 ± 3.9	0.75	0.0029

^1^ Standard error of the mean. ^2^ Differences considered significant at *p* < 0.05 and tending towards significance at 0.05 < *p* ≤ 0.10.

## Data Availability

Not applicable.

## Supplementary Materials

The following are available online at https://www.mdpi.com/article/10.3390/ani12202830/s1. Table S1: Definitions of the sensory attributes tested. Table S2: Individual data on technological meat quality attributes of fresh meat (colour, cooking loss and WBSF) and sensory attributes (Small = small-scale abattoir, Large = large-scale abattoir). Table S3: Individual data on technological meat quality attributes of frozen meat (colour, cooking loss and WBSF) and sensory attributes (Small = small-scale abattoir, Large = large-scale abattoir). Table S4: Translational relaxation parameters and water population within muscle samples from fresh meat (Small = small-scale abattoir, Large = large-scale abattoir). Table S5: Translational relaxation parameters and water population within muscle samples from frozen meat (Small = small-scale abattoir, Large = large-scale abattoir).
