# Supplementary material for: A Comparison of Fresh and Frozen Lamb Meat—Differences in Technological Meat Quality and Sensory Attributes
Source: Animals (Basel). 2022 Oct 18;12(20):2830. doi: 10.3390/ani12202830 (PMC9597754; doi:10.3390/ani12202830)
Supplement: Supplementary file 1 [file animals-12-02830-s001.zip › animals-1951113-supplementary.pdf]

Supplementary data

Table S1. Definitions of the sensory attributes tested.

<table>
<tr><th>Sensory attribute</th><th>Short name</th><th>Scale</th><th>Definition</th></tr>
<tr><td>ODOUR</td><td></td><td></td><td></td></tr>
<tr><td>Frying</td><td>O-frying</td><td>none | | much</td><td>Odour of fryed lamb meat</td></tr>
<tr><td>Sour</td><td>O-sour</td><td>none | | much</td><td>Sour odour of fresh lamb meat</td></tr>
<tr><td>Fatty</td><td>O-fatty</td><td>none | | much</td><td>Lamb fat, wool</td></tr>
<tr><td>Liver</td><td>O-liver</td><td>none | | much</td><td>Odour of lamb liver, blood, iron, game</td></tr>
<tr><td>APPEARANCE</td><td></td><td></td><td></td></tr>
<tr><td>Colour</td><td>A-colour</td><td>light | | dark</td><td>Colour of sample´s cutted surface</td></tr>
<tr><td>FLAVOUR</td><td></td><td></td><td></td></tr>
<tr><td>Frying</td><td>F-frying</td><td>none | | much</td><td>Flavour of fryed lamb meat</td></tr>
<tr><td>Sour</td><td>F-sour</td><td>none | | much</td><td>Sour flavour of fresh lamb meat</td></tr>
<tr><td>Fatty</td><td>F-fatty</td><td>none | | much</td><td>Flavour of lamb fat, wool</td></tr>
<tr><td>Sweet</td><td>F-sweet</td><td>none | | much</td><td>Sweet flavour</td></tr>
<tr><td>Liver</td><td>F-liver</td><td>none | | much</td><td>Flavour of lamb liver, blood, iron, game</td></tr>
<tr><td>TEXTURE</td><td></td><td></td><td></td></tr>
<tr><td>Soft</td><td>T-soft</td><td>firm | | soft</td><td>First bite, cut a piece from the middle of the sample and bite transversely on the muscle fibers</td></tr>
<tr><td>Tender</td><td>T-tender</td><td>tough | | tender</td><td>How tough or tender is the meat when chewing</td></tr>
<tr><td>Juicy</td><td>T-juicy</td><td>dry | | juicy</td><td>Dry - draws juice from mouth; juicy- releases juice</td></tr>
<tr><td>Mushy</td><td>T-mushy</td><td>none | | much</td><td>Mushy, lamb paté</td></tr>
</table>

Table S2. Fresh meat, with individual data on technological meat quality attributes (colour, cooking loss and WBSF) and sensory attributes (Small = Small-scale slaughter system, Large = Large-scale slaughter system).

Slaughter system	Small	Small	Small	Small	Small	Small	Small	Small	Small	Small
Animal ID	1189	1581	1685	1465	1533	1812	1570	1389	1699	1872
Lamb pair	1	2	3	4	5	6	7	8	9	10
L*	37.0	38.1	36.2	35.2	34.0	36.3	37.2	35.4	39.3	36.7
a*	19.7	18.8	20.8	20.4	18.4	15.1	17.4	20.8	15.6	21.1
b*	5.3	3.9	5.1	3.6	2.8	2.3	3.2	6.2	3.3	5.9
Cooking loss (%)	14.2	17.4	9.3	16.7	11.9	9.3	19.8	14.4	12.0	13.7
WBSF (N)[a]	87.2	39.6	49.7	38.4	54.0	46.9	56.6	43.4	41.8	32.8
Frying odour[b]	25	30	43	35	29	33	30	46	29	30
Sour odour[b]	9	17	17	9	22	13	11	11	12	8
Fatty odour[b]	28	28	23	31	30	29	22	28	27	29
Liver odour[b]	31	29	30	32	33	25	23	29	24	38
Colour apperance[b]	30	30	42	26	35	31	34	32	34	38
Frying flavour[b]	24	25	26	21	27	23	24	38	27	22
Sour flavour[b]	17	23	25	31	25	28	28	16	26	25
Fatty flavour[b]	17	17	16	14	22	13	18	22	23	17
Sweet flavour[b]	8	9	11	9	7	10	8	10	8	9
Liver flavour[b]	42	47	50	45	37	36	34	38	35	42
Soft texture[b]	40	54	66	28	35	45	36	66	34	69
Tender texture[b]	36	51	63	26	29	53	29	69	29	71
Juicy texture[b]	49	37	60	39	34	54	42	67	42	62
Muschy texture[b]	12	14	15	10	13	19	11	16	12	22
Slaughter system	Large	Large	Large	Large	Large	Large	Large	Large	Large	Large
Animal ID	2812	2699	2872	2685	2389	2533	2189	2465	2570	2581
Lamb pair	6	9	10	3	8	5	1	4	7	2

L*	37.0	40.1	.	39.1	36.4	.	37.5	39.9	38.8	39.0
a*	20.9	19.5	.	20.4	21.3	.	20.2	18.1	19.5	20.7
b*	4.1	5.3	.	5.9	5.2	.	3.9	4.6	4.3	5.5
Cooking loss (%)	18.2	18.5	.	11.8	17.4	.	14.7	15.1	13.3	16.1
WBSF (N)[a]	32.6	37.4	.	49.5	48.3	.	50.7	49.5	43.8	25.5
Frying odour[b]	22	55	.	24	35	.	30	40	36	21
Sour odour[b]	15	10	.	9	17	.	9	12	13	11
Fatty odour[b]	28	25	.	28	31	.	20	32	29	26
Liver odour[b]	35	32	.	29	20	.	27	34	33	30
Colour apperance[b]	27	30	.	34	25	.	27	36	29	25
Frying flavour[b]	20	31	.	22	23	.	20	25	24	19
Sour flavour[b]	30	32	.	19	22	.	19	31	27	32
Fatty flavour[b]	15	18	.	11	15	.	11	14	19	17
Sweet flavour[b]	11	13	.	7	9	.	7	9	8	10
Liver flavour[b]	47	47	.	35	40	.	35	46	44	39
Soft texture[b]	64	64	.	38	47	.	46	48	44	72
Tender texture[b]	58	69	.	34	43	.	45	42	43	72
Juicy texture[b]	57	51	.	38	47	.	50	49	46	62
Muschy texture[b]	19	24	.	11	13	.	17	13	16	26

[a]Warner-Bratzler shear force measured in Newton.
[b]Sensory attributes was scored on a scale from 0-100, with 100 being the highest score.

Table S3. Frozen meat, individual data on technological meat quality attributes (colour, cooking loss and WBSF) and sensory attributes (Small = Small-scale slaughter system, Large = Large-scale slaughter system).

Slaughter system	Small	Small	Small	Small	Small	Small	Small	Small	Small	Small
Animal ID	1189	1581	1685	1465	1533	1812	1570	1389	1699	1872
Lamb pair	1	2	3	4	5	6	7	8	9	10
L*	36.0	36.0	35.9	37.5	36.9	35.7	38.4	35.6	37.0	36.6
a*	19.7	18.2	22.0	20.2	18.8	16.5	16.5	18.8	18.2	21.5
b*	6.7	7.0	7.0	8.2	6.7	4.7	3.4	4.1	5.9	7.5
Cooking loss (%)	26.7	29.9	22.2	26.2	28.2	23.1	32.7	23.6	18.2	27.7
WBSF (N)[a]	73.6	40.2	38.9	30.9	56.8	73.7	58.3	27.1	46.6	30.2
Frying odour[b]	31	41	33	34	43	27	29	31	29	45
Sour odour[b]	16	13	15	16	10	15	12	20	13	11
Fatty odour[b]	31	27	30	31	26	37	35	39	32	25
Liver odour[b]	32	34	35	26	26	31	37	33	23	34
Colour apperance[b]	37	19	35	30	31	40	31	32	36	38
Frying flavour[b]	25	32	30	21	30	30	26	20	20	34
Sour flavour[b]	15	36	29	27	28	27	3	43	19	25
Fatty flavour[b]	21	17	23	21	18	33	29	19	26	23
Sweet flavour[b]	13	11	10	10	9	6	10	8	14	8
Liver flavour[b]	36	44	44	55	35	48	46	44	45	48
Soft texture[b]	52	62	44	72	36	33	47	64	70	58
Tender texture[b]	45	60	42	70	32	28	46	64	69	55
Juicy texture[b]	26	34	43	48	23	26	30	45	56	36
Muschy texture[b]	17	12	10	11	9	6	14	21	21	15
Slaughter system	Large	Large	Large	Large	Large	Large	Large	Large	Large	Large
Animal ID	2812	2699	2872	2685	2389	2533	2189	2465	2570	2581
Lamb pair	6	9	10	3	8	5	1	4	7	2
L*	37.9	38.2	36.0	36.6	34.7	34.0	37.1	38.2	36.5	39.8
a*	19.0	18.2	19.3	18.9	18.6	19.5	19.1	17.0	17.4	18.3
b*	6.8	7.1	5.9	5.6	5.0	5.0	6.7	6.3	2.9	7.3
Cooking loss (%)	27.7	29.3	30.3	25.2	28.5	29.6	25.6	27.2	26.8	28.6

WBSF (N)[a]	42.6	55.5	57.3	57.3	52.3	65.3	55.6	50.4	54.4	46.3
Frying odour[b]	38	38	44	37	28	37	39	31	33	50
Sour odour[b]	18	17	9	16	13	16	11	14	12	17
Fatty odour[b]	28	36	28	27	46	24	29	23	49	28
Liver odour[b]	28	30	24	36	31	37	37	36	33	33
Colour apperance[b]	24	24	30	36	22	38	24	19	37	23
Frying flavour[b]	33	26	40	35	22	21	31	25	24	45
Sour flavour[b]	36	38	28	27	37	21	41	46	28	36
Fatty flavour[b]	27	19	16	22	19	16	17	17	24	27
Sweet flavour[b]	8	10	9	10	8	9	9	9	8	8
Liver flavour[b]	48	48	42	46	36	37	43	51	49	48
Soft texture[b]	75	45	54	43	32	36	49	47	51	75
Tender texture[b]	72	44	49	40	32	30	41	40	58	72
Juicy texture[b]	52	32	34	29	20	21	25	25	42	52
Muschy texture[b]	16	12	15	13	13	11	13	10	14	16

[a]Warner-Bratzler shear force measured in Newton.
[b]Sensory attributes was scored on a scale from 0-100, with 100 being the highest score.

Table S4. **Tr**anslational relaxation parameters and water population within muscle samples from fresh meat (Small = Small-scale slaughter system, Large = Large-scale slaughter system).

Slaughter system	Small	Small	Small	Small	Small	Small	Small	Small	Small	Small
Animal ID	1189	1581	1685	1465	1533	1812	1570	1389	1699	1872
Lamb pair	1	2	3	4	5	6	7	8	9	10
T2b[a]	24.1	23.6	8.3	16.9	11.9	13.1	19.7	8.6	8.5	11.0
T21[a]	52.2	56.0	45.3	50.7	44.2	45.3	51.5	46.4	46.7	51.0
T22[a]	313.1	379.6	182.3	236.4	169.0	232.7	267.5	166.5	156.8	173.0
A2b[b]	19.0	15.4	4.3	14.6	4.8	6.8	16.9	3.6	4.1	5.1
A21[b]	73.0	76.4	86.4	73.8	87.4	85.2	72.5	86.5	84.1	82.4
A22[b]	8.0	8.2	9.3	11.6	7.9	8.0	10.5	10.0	11.7	12.4
Slaughter system	Large	Large	Large	Large	Large	Large	Large	Large	Large	Large
Animal ID	2812	2699	2872	2685	2389	2533	2189	2465	2570	2581
Lamb pair	6	9	10	3	8	5	1	4	7	2
T2b[a]	18.8	5.9	.	28.6	5.1	.	12.6	10.5	14.2	5.0
T21[a]	53.9	46.7	.	58.4	44.3	.	47.7	44.2	48.4	44.3
T22[a]	312.2	143.6	.	432.6	127.0	.	221.3	178.4	251.4	97.1
A2b[b]	9.2	3.4	.	30.9	3.2	.	9.1	4.3	7.5	3.0
A21[b]	85.5	86.0	.	60.8	85.2	.	82.8	88.3	85.0	83.8
A22[b]	5.3	10.6	.	8.3	11.6	.	8.1	7.4	7.5	13.2

[a]Translational relaxation parameters.
[b]Water population within muscle sample.

Table S5. **Tr**anslational relaxation parameters and water population in muscle samples after freezing (Small = Small-scale slaughter system, Large = Large-scale slaughter system).

Slaughter system	Small	Small	Small	Small	Small	Small	Small	Small	Small	Small
Animal ID	1189	1581	1685	1465	1533	1812	1570	1389	1699	1872
Lamb pair	1	2	3	4	5	6	7	8	9	10
T2b[a]	5.2	7.7	16.1	.	.	22.4	16.5	11.0	6.4	14.4
T21[a]	37.5	41.5	42.2	.	.	42.2	37.2	37.0	40.1	43.7
T22[a]	89.1	96.4	130.3	.	.	108.3	81.3	71.2	98.9	131.5
A2b[b]	3.5	3.3	8.9	.	.	16.1	9.2	2.8	3.5	6.8
A21[b]	82.6	83.0	81.4	.	.	76.8	74.7	74.4	83.5	79.9

A22[b]	14.0	13.7	9.7	.	.	7.1	16.1	22.7	12.9	13.3
Slaughter system	Large	Large	Large	Large	Large	Large	Large	Large	Large	Large
Animal ID	2812	2699	2872	2685	2389	2533	2189	2465	2570	2581
Lamb pair	6	9	10	3	8	5	1	4	7	2
T2b[a]	5.3	25.1	11.3	6.7	36.6	9.8	2.7	10.9	7.4	2.1
T21[a]	42.1	44.1	44.1	40.1	54.6	41.7	34.5	39.3	38.7	38.2
T22[a]	91.9	129.6	132.6	120.3	118.0	127.9	75.5	114.1	100.6	89.8
A2b[b]	2.8	14.6	5.1	4.0	65.1	5.5	3.3	4.6	3.4	3.4
A21[b]	80.8	77.6	83.5	82.7	26.5	82.0	80.3	85.3	86.3	80.3
A22[b]	16.4	7.8	11.4	13.3	8.4	12.5	16.4	10.1	10.3	16.3

[a]Translational relaxation parameters.

[b]Water population within muscle sample.
